# Potential for Aerobic Methanotrophic Metabolism on Mars

**DOI:** 10.1089/ast.2018.1943

**Published:** 2019-10-03

**Authors:** Mayumi Seto, Katsuyuki Noguchi, Philippe Van Cappellen

**Affiliations:** ^1^ Department of Chemistry, Biology, and Environmental Sciences, Faculty of Science, Nara Women's University, Nara, Japan.; ^2^ Ecohydrology Research Group, Department of Earth and Environmental Sciences, Water Institute, University of Waterloo, Waterloo, Canada.

**Keywords:** Mars, Methane, Methanotrophs, Catabolic energy production, Maintenance energy demand, Kinetic limitation

## Abstract

Observational evidence supports the presence of methane (CH_4_) in the martian atmosphere on the order of parts per billion by volume (ppbv). Here, we assess whether aerobic methanotrophy is a potentially viable metabolism in the martian upper regolith, by calculating metabolic energy gain rates under assumed conditions of martian surface temperature, pressure, and atmospheric composition. Using kinetic parameters for 19 terrestrial aerobic methanotrophic strains, we show that even under the imposed low temperature and pressure extremes (180–280 K and 6–11 hPa), methane oxidation by oxygen (O_2_) should in principle be able to generate the minimum energy production rate required to support endogenous metabolism (*i.e.*, cellular maintenance). Our results further indicate that the corresponding metabolic activity would be extremely low, with cell doubling times in excess of 4000 Earth years at the present-day ppbv-level CH_4_ mixing ratios in the atmosphere of Mars. Thus, while aerobic methanotrophic microorganisms similar to those found on Earth could theoretically maintain their vital functions, they are unlikely to constitute prolific members of hypothetical martian soil communities.

## 1. Introduction

Mars is considered a promising planet in the quest for extraterrestrial life because of its resemblance to Earth and the accumulating evidence of past and potentially current surface and subsurface water activity (Pollack and Kasting, 1987; McKay *et al.*, 1996; Squyres *et al.*, 2004; Orosei *et al.*, 2018). At present, however, life would be challenged by the environmental conditions on Mars, including the scarcity of liquid water, very low atmospheric pressure and extreme temperature variations, a carbon dioxide (CO_2_)-dominated atmosphere, strong UV and cosmic radiation, high concentrations of heavy metals, the lack of organic substances and nutrients, and high pH (Berry *et al.*, 2010). Nonetheless, the fact that microorganisms survive in extreme environments on Earth (Junge *et al.*, 2004; Berry *et al.*, 2010; Nicholson *et al.*, 2013; Moissl-Eichinger *et al.*, 2016) supports the possibility of microbial life under the harsh conditions found on Mars.

Based on a re-evaluation of the environmental limits for microbial life, Rummel *et al.* (2014) identified *Special Regions* on Mars within which microorganisms similar to those found on Earth could survive and propagate. Life forms best adapted to the martian conditions are likely chemolithoautotrophic microorganisms that use inorganic carbon sources to build new biomass and generate energy from the oxidation of inorganic compounds. Any microbial cell requires a minimum energy production to support basic metabolic functions and activity in the absence of growth, the so-called maintenance energy requirement (Hoehler, 2004). Only when the catabolic energy production exceeds the maintenance energy demand, a cell becomes able to allocate the remaining energy to reproduction. To determine whether this threshold is reached for a given group of chemolithotrophs, one should thus compare the potential catabolic energy production in a given environment with the cellular maintenance requirement.

Here, we assess the minimum criteria for survival of aerobic methanotrophic microorganisms by estimating their catabolic energy production under assumed martian near-surface conditions. On Earth, aerobic methanotrophs are widely distributed in soils, sediments, and aqueous ecosystems, and some of them have been shown to be tolerant toward various environmental stressors (Hanson and Hanson, 1996; Trotsenko and Khmelenina, 2002, 2005). These microorganisms utilize CH_4_ as the electron donor and O_2_ as terminal electron acceptor to generate energy, according to the overall catabolic reaction:

\begin{align*}
{ \rm{C}}{{ \rm{H}}_{ \rm{4}}}{ \rm{ + 2}}{{ \rm{O}}_{ \rm{2}}} \rightharpoonup { \rm{C}}{{ \rm{O}}_{ \rm{2}}}{ \rm{ + 2}}{{ \rm{H}}_{ \rm{2}}}{ \rm{O}}. \tag{1}
\end{align*}

Many aerobic microorganisms are able to grow at relatively low O_2_ concentrations, similar to those encountered in the martian atmosphere (on average 0.13% by volume, Rummel *et al.*, 2014). The extreme low abundance of CH_4_ may therefore represent a more severe limitation for aerobic methanotrophs on Mars. On Earth, some methanotrophic microbes have been found to possess a “high-affinity” enzyme for CH_4_ (Conrad, 1996; Hanson and Hanson, 1996), which allows them to function at ppmv levels of CH_4_ in well-aerated upper soil horizons (Knief and Dunfield, 2005). Nonetheless, the current mixing ratio of CH_4_ in Earth's atmosphere (approximately 1.75 ppmv, Conrad, 1996; Bull *et al.*, 2000) is two to four orders of magnitude higher than current estimates for the martian atmosphere (Formisano *et al.*, 2004; Krasnopolsky *et al.*, 2004; Mumma *et al.*, 2009; Fonti and Marzo, 2010; Webster *et al.*, 2013, 2015, 2018). The possible sources of martian CH_4_ include exogenous inputs, for example, from meteorite impacts, hydrothermal processes, for example, serpentinization reactions, and biological emissions (Atreya *et al.*, 2007).

At first sight, the very low levels of CH_4_ in the martian atmosphere would seem to preclude aerobic methanotrophy. Nevertheless, cells on Mars may have much lower maintenance energy requirements than most of their terrestrial counterparts because of the very low surface temperatures. On Earth, microbial communities found at subfreezing temperatures are able to survive by greatly reducing their energy expenditures (Price and Sowers, 2004). For example, immobile or dormant nitrifiers remain viable at −40°C due to their extremely slow metabolism and, hence, very low maintenance energy requirements (Sowers, 2001; Price and Sowers, 2004).

In this article, we begin by outlining the theoretical basis for estimating cell-specific rates of catabolic energy production and maintenance energy consumption by methanotrophic microorganisms. We then derive the equations for (1) the minimum CH_4_ concentration required to support cell maintenance, and (2) cell doubling times. The latter provides an indicator for the potential for growth of the microbial population. Next, we calculate the metabolic energy gain rates under near-surface martian conditions using kinetic parameters extracted from 19 known terrestrial methanotrophic strains, and discuss the uncertainties associated with the estimations and the implications for methanotrophy on Mars.

## 2. Methods

### 2.1. Catabolic energy production at given temperature, pressure, and mixing ratios

The cell-specific rate of catabolic energy production by methanotrophs depends on the Gibbs energy change of the CH_4_ oxidation reaction by O_2_ [Eq. (1)] (McCollom and Shock, 1997; Amend and Shock, 2001; Shock *et al.*, 2010):

\begin{align*}
{ \Delta _r } G = { \Delta _r } { G^\circ } + RT { \rm { ln } } { \frac { { f_ { { \rm { C } } { { \rm { O } } _2 } } } }  { f_ { { { \rm { O } } _2 } } ^2\, { f_ { { \rm { C } } { { \rm { H } } _4 } } } } } , \tag { 2 }
\end{align*}

where Δ*_r_G* is expressed in kJ (mol CH_4_)^−1^, *R* is the gas constant in units of kJ K^−1^ mol^−1^, *T* is the absolute temperature in K, *f_X_* stands for the fugacity of a chemical species *X*, and Δ*_r_G*° is the standard Gibbs free energy change of the reaction at a given temperature and pressure in units of kJ (mol CH_4_)^−1^:

\begin{align*}
{ \Delta _r}{G^ \circ } = { \Delta _f}G_{{ \rm{C}}{{ \rm{O}}_2}}^ \circ + 2{ \Delta _f}G_{{{ \rm{H}}_2}{ \rm{O}}}^ \circ - { \Delta _f}G_{{ \rm{C}}{{ \rm{H}}_4}}^ \circ - 2{ \Delta _f}G_{{{ \rm{O}}_2}}^ \circ , \tag{3}
\end{align*}

where Δ*_f_G_X_*° is the standard molal Gibbs free energy of formation of a species *X* in units of kJ mol^−1^. At a given temperature and pressure, *T_g_* and *P_g_*, the Δ*_f_G*° values are calculated following the work of Helgeson *et al.* (1978):

\begin{align*}
\begin{split} { \Delta _f}{G^ \circ } = { \Delta _f}{G^{ \circ *}} - {S^{ \circ *}} \left( {{T_g} - {T^*}} \right) + \mathop \int \limits_{{T^*}}^{{T_g}} C_P^{ \circ *} \;dT \\- \mathop \int \limits_{{T^*}}^{{T_g}} C_P^{ \circ *} \;d{ \rm{ln}}T + \mathop \int \limits_{{P^*}}^{{P_g}} V_{}^{ \circ *} \;dP ,\end{split}
\tag{4}
\end{align*}

where superscript * indicates reference values at 298.15 K and/or 1 bar, while *S*° is the standard partial molal entropy at the reference conditions in units of kJ mol^−1^, and *C_P_*° and *V*° designate the standard partial molal isobaric heat capacity in units of kJ K^−1^ mol^−1^ and the standard partial molal isobaric volume, respectively. The following empirical equation for the standard molal heat capacity in units of kJ mol^−1^ K^−1^ at 1 bar is substituted into Eq. (4):

\begin{align*}
{C_P}^{\circ *}  / R = A{ \rm{ }} + { \rm{ }}BT - C{T^2} , \tag{5}
\end{align*}

where *A*, *B*, and *C* are experimentally derived, temperature-independent coefficients for the species of interest (Supplementary Table S1). For gaseous species, *V*°* = *RT*/*P* in units of kJ Pa^−1^ mol^−1^, and we further assume that *V*°* of water is constant (18 × 10^−6^ m^3^ mol^−1^ = 18 × 10^−9^ kJ Pa^−1^ mol^−1^), and that fugacities, *f_X_*, obey van der Waals equation:

\begin{align*}
{ f_X } = { \gamma _X } { P_ { tot } } { \rm { \;exp } } \left\{ { { \frac { { \gamma _X } \; { P_ { tot } } }  { RT } } \left( { b - \frac { a }  { { RT } } } \right) } \right\} , \tag { 6 }
\end{align*}

where *γ_X_* is the concentration of a gaseous species *X* in (v/v), *P_tot_* denotes the total pressure (atm), and *a* and *b* are the van der Waals constants (Supplementary Table S1).

The standard formalism to describe rates of microbially catalyzed reaction processes is the Michaelis–Menten kinetic equation. For CH_4_ oxidation, the cell-specific rate is as follows:

\begin{align*}
r = { v_ { max } } { \frac { \left[ { { \rm { C } } { { \rm { H } } _4 } } \right] }  { { K_ { { \rm { C } } { { \rm { H } } _4 } } } + \left[ { { \rm { C } } { { \rm { H } } _4 } } \right] } } { \frac { \left[ { { { \rm { O } } _2 } } \right] }  { { K_ { { { \rm { O } } _2 } } } + \left[ { { { \rm { O } } _2 } } \right] } } , \tag { 7 }
\end{align*}

where *v_max_* is the maximum cell-specific CH_4_ oxidation rate (mol CH_4_ cell^−1^ h^−1^), *K_X_* are half-saturation constants (mol L^−1^), and [CH_4_] and [O_2_] are the molar concentrations of CH_4_ and O_2_ (mol L^−1^), which are obtained from the following:

\begin{align*}
\left[ X \right] = { \frac { { \gamma _X } { P_ { tot } } }  { RT } } , \tag { 8 }
\end{align*}

where the gas constant, *R*, is expressed in units of L atm K^−1^ mol^−1^. To calculate cell-specific CH_4_ oxidation rates with Eq. (7), values of *v_max_* and *K*_CH4_ for 19 strains of aerobic methanotrophs were extracted from the literature (Knief and Dunfield, 2005). The corresponding *v_max_* values range from 5.6 × 10^−18^ to 5.7 × 10^−16^ mol CH_4_ cell^−1^ h^−1^, and *K*_CH4_ from 1.0 × 10^−6^ to 2.3 × 10^−5^ mol L^−1^. For *K*_O2_, the only reported value is that for *Methylocystis* sp. strain *MOX-1* (1.9 × 10^−9^ mol L^−1^; Van Bodegom *et al.*, 2001). This value is therefore applied in all the following calculations.

The cell-specific catabolic energy production rate, which is equivalent to the amount of power available to the microorganisms (*i.e.*, the “power supply,” LaRowe and Amend, 2015), is calculated by multiplying the Gibbs energy change of reaction of aerobic CH_4_ oxidation with the cell-specific CH_4_ oxidation rate:

\begin{align*}
\left[ {{ \rm{Cell{\hbox -}specific \ catabolic \ energy \ production \ rate}}} \right] { \rm{ \  = }} \  -   r \bullet { \Delta _r}G , \tag{9}
\end{align*}

### 2.2. Metabolic energy requirement of endogenous metabolism

Temperature is a key environmental control on cellular energy requirements for growth, maintenance, and survival (Tijhuis *et al.*, 1993; Price and Sowers, 2004). Tijhuis *et al.* (1993) derived empirical correlations between the maintenance energy demand rate, expressed in kJ (mol C biomass)^−1^ h^−1^, and temperature for both aerobic and anaerobic bacteria in the temperature range of 2–66°C. This temperature range, however, does not encompass the subzero martian surface temperatures.

**FIG. 1.**
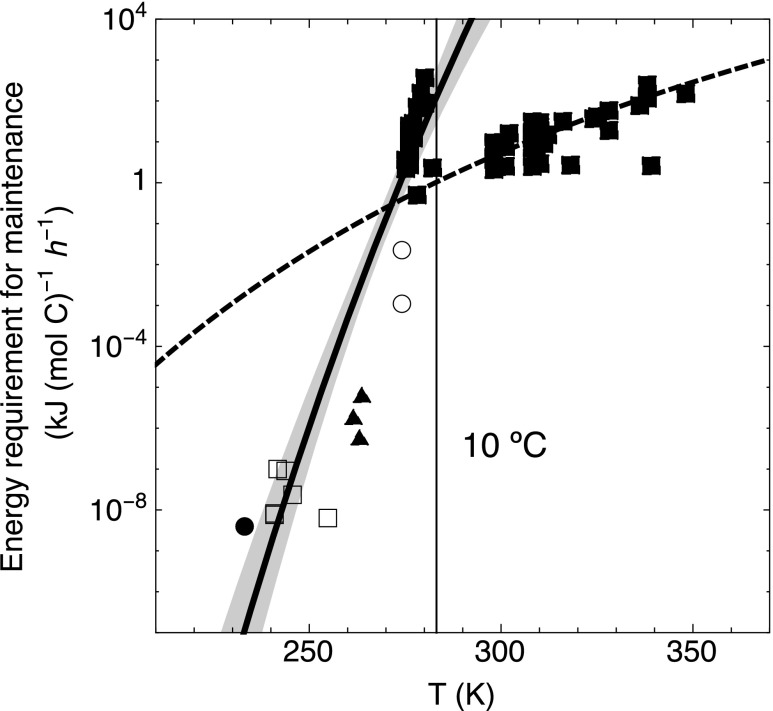
Correlations between temperature and maintenance energy using data of aerobic and anaerobic bacteria shown as filled squares (Tijhuis *et al.*, 1993), methanogens shown as open circles (Price and Sowers, 2004) and filled triangles (Tung *et al.*, 2005), and nitrifiers shown as open squares (Rohde *et al.*, 2008) and a filled circle (Price and Sowers, 2004) (Supplementary Table S2). The dashed line is the correlation reported by Tijhuis *et al.*, and the black line is our correlation for temperatures below 10°C: ${ m_E } { \rm { = 1 } } { { \rm { 0 } } ^ { - { \rm { 15 } } } } { \rm { exp } } \left( { { \rm { 144 } } { \rm { .9 } } - { \rm { } } \frac { { { \rm { 39 , 656 } } } }  { T } } \right)$. Light shading indicates the 95% confidence level.

The existing, admittedly limited, data indicate that the temperature dependence of cellular metabolic energy requirements differs between mesophilic and psychrophilic or psychrotolerant microorganisms, that is, microorganisms growing and metabolically active at temperatures of 0°C and below. This is shown in Fig. 1, where the estimated metabolic energy requirement rates for maintenance and survival of nitrifiers and methanogens at temperatures between −40°C and 0°C (Price and Sowers, 2004; Tung *et al.*, 2005; Rohde *et al.*, 2008; Supplementary Table S2) are compared with the data for aerobic and anaerobic bacteria of Tijhuis *et al.* (1993). Note that the subzero maintenance energy requirements are extracted from the original article figures using the Plot Digitizer software (available at http://plotdigitizer.sourceforge.net). The original units of gC (gC)^−1^ h^−1^ are converted to kJ (mol C)^−1^ h^−1^ by considering the Gibbs energy needed to synthesize 1 gC of biomass, which for autotrophic nitrifiers is 291.4 kJ gC^−1^, and for heterotrophs that utilize organic carbon source as their electron donor is estimated by using the empirical equation of Heijnen (2010):

\begin{align*}
Y =  \bigg (  {200 + 18{{ \big ( {6 - C} \big ) }^{1.8}}  + { \rm{exp}} \left[ {{{ \left\vert {3.8 - \gamma } \right\vert }^{0.32}} \times \left( {3.6 + 0.4C} \right) } \right] }  \bigg ) / 12.01 , \tag{10}
\end{align*}

where *C* is the number of C-atoms of a given carbon source and *γ* is the degree of reduction of the C atoms in the carbon source.

As seen in Fig. 1, the trend of the maintenance energy demand at temperatures below 10°C diverges markedly from that for temperatures above 10°C, hence suggesting that the dependence of the metabolic energy requirement of psychrophiles (or psychrotolerants) is not captured by the Tijhuis *et al.* (1993) equation. The low maintenance energy requirement rates likely represent an adaptive response where cells facing severe temperature stress reduce their endogenous metabolism to the most essential functions only (Kempes *et al.*, 2017).

The temperature-dependent metabolic energy requirement rate in kJ (mol C)^−1^ h^−1^ at cold temperatures is obtained by fitting the ≤10°C data in Fig. 1 to an Arrhenius-type equation. Assuming a carbon content of methanotrophic cells of 12 fg-C cell^−1^ (Nihous and Masutani, 2007), the cell-specific endogenous metabolic energy requirement rate in units of kJ cell^−1^ h^−1^, *m_E_*, is then as follows:

\begin{align*}
{ m_E } { \rm { = 1 } } { { \rm { 0 } } ^ { - { \rm { 15 } } } } { \rm { \;exp } } \left( { { \rm { 144 } } { \rm { .9 } } - { \rm { } } \frac { { { \rm { 39 , 656 } } } }  { T } } \right) , \tag { 11 }
\end{align*}

where *T* is expressed in K. In the following, Eq. (11) is used to estimate the metabolic requirements of aerobic methanotrophs under martian surface temperatures. The cell-specific net energy gain rate is further obtained by subtracting *m_E_* from the cell-specific rate of catabolic energy production given by Eq. (9):

\begin{align*}
\left[ {{ \rm{Cell{\hbox -}specific \ net \ energy \ gain \ rate}}} \right] { \rm{ \; = }} -   r \bullet { \Delta _r}G - {m_E}. \tag{12}
\end{align*}

The cell-specific net energy gain rate is a proxy measure of the potential for a given group of microorganisms to survive in a given environment. It is also referred to as the organisms' invasibility according to the definition of Seto (2014). When the net energy gain rate is positive, the microorganisms can, in principle, invest the energy in excess to that required for their maintenance functions into growth. By letting Eq. (12) be 0 and solving for the corresponding CH_4_ concentration, we can calculate the minimum CH_4_ requirement for aerobic methanotrophs to survive under the assumed martian conditions.

### 2.3. Cell doubling times

Assuming exponential growth, the cell doubling time is simply the inverse of the specific growth rate multiplied by ln 2. The specific growth rate in turn is obtained by dividing the cell-specific net energy gain rate by the Gibbs energy required to synthesize 1 cell, *Y* (kJ cell^−1^). The cell doubling time is thus given by the following:

\begin{align*}
\left[ { { \rm { Cell \ doubling \ time } } } \right] { \rm { = } } { \frac { Y \ln 2 }  { - r { \Delta _r } G - { m_E } } } . \tag { 13 }
\end{align*}

The value of *Y* is computed with Eq. (10) assuming CH_4_ is the sole carbon source. This gives *Y* = 1.086 × 10^−12^ kJ cell^−1^. Note that Eq. (12) does not account for the energy dissipated as heat, and therefore, Eq. (13) yields a minimum estimate of the cell doubling time.

## 3. Results

### 3.1. Thermodynamics and kinetics of aerobic methane oxidation on Mars

Values of Δ*_r_G* and the cell-specific microbial CH_4_ oxidation rate, *r*, are calculated for the ranges in temperature and pressure encountered at the surface of Mars (180–280 K, 6–11 hPa; Martínez *et al.*, 2017), while fixing the mixing ratios of CO_2_ and O_2_ to 95% and 0.145%, respectively (Haberle *et al.*, 2017). The default mixing ratio of CH_4_ is set to 0.69 parts per billion by volume (ppbv), which is the average of background levels of atmospheric CH_4_ reported for *in situ* measurements made with the tunable laser spectrometer on Mars' Curiosity rover (Webster *et al.*, 2015).

Terrestrial-like microbial cells could not survive exposed at the surface of Mars because of the intense UV radiation and ionizing radiation. Rather, they would be expected to reside below the surface shielded by at least 1 mm of regolith to avoid UV radiation (Rummel *et al.*, 2014) and at least 20 cm to avoid ionizing radiation (Dartnell, 2011). Methanotrophy would then rely on O_2_ produced photochemically in the atmosphere and CH_4_ released from source areas presumably located at depths of several km (Stevens *et al.*, 2015). In our calculations, we assume that the CH_4_ soil gas concentration in the upper martian regolith equals that measured in the atmosphere (0.69 ppbv). This is reasonable in view of the low CH_4_ outgassing flux of 3.6 × 10^−19^ mol CH_4_ cm^−2^ s^−1^ estimated by Krasnopolsky *et al.* (2004), which, together with typical values for gas diffusion coefficients, yields vertical CH_4_ concentration gradients of the order of 10^−18^ mol cm^−4^. In other words, within the uppermost meters of regolith, the fugacity of CH_4_ should be close to that in the overlying atmosphere.

**FIG. 2.**
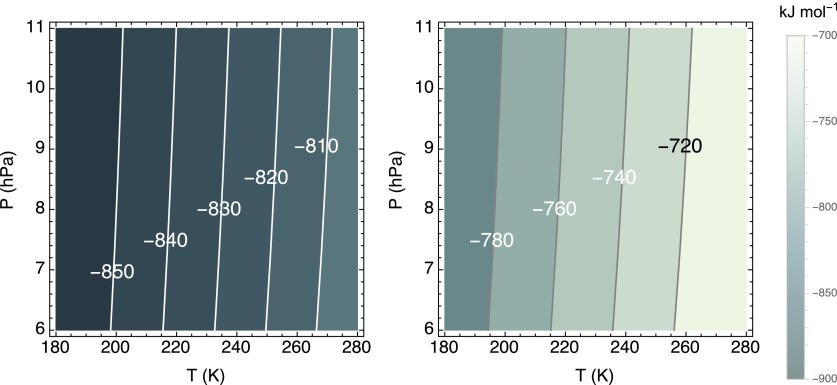
Standard Gibbs free energy change, Δ*_r_G*°, and the Gibbs free energy change, Δ*_r_G*, of methane oxidation (left and right panel, respectively) in units of kJ (mol CH_4_)^−1^ against temperature and pressure. The imposed mixing ratios of CO_2_, O_2_, and CH_4_ are 95%, 0.145%, and 0.69 ppbv, respectively.

For the gas mixing ratios quoted above, and at the usual reference temperature and pressure of 298.13 K and 1 atm, Equation (1) is highly exergonic, with a Δ*_r_G* value of −733.3 kJ (mol CH_4_)^−1^ [note: the corresponding Δ*_r_G*° equals −817.9 kJ (mol CH_4_)^−1^]. Under martian surface conditions, the calculated Gibbs energies of reaction are of the same order of magnitude (Fig. 2), implying that the low temperatures and pressures on Mars do not significantly alter the thermodynamic energy yield of the aerobic methane oxidation reaction. Even at the very low inferred CH_4_ concentrations, Equation (1) remains thermodynamically highly favorable; CH_4_ concentrations two orders of magnitude higher than 0.69 ppbv only yield Δ*_r_G* values that are around 10 kJ (mol CH_4_)^−1^ more negative at 230 K and 7 hPa (results not shown).

The resulting cell-specific CH_4_ oxidation rates calculated with Eq. (7) fall well below the maximum rates *v_max_*, that is, they are strongly limited by substrate availability, in particular the low availability of CH_4_. For comparison, when the mixing ratio of CH_4_ is set to the Earth's atmosphere average of 1.8 ppm, the calculated methane oxidation rates are four orders of magnitude faster than those calculated for the default martian CH_4_ mixing ratio of 0.69 ppbv, all other conditions equal. The predicted limitation of martian CH_4_ oxidation kinetics is a direct consequence of the relative magnitudes of the concentrations of the gaseous reactants and the corresponding half-saturation constants in the Michaelis–Menten formulation of Eq. (7). For the 19 terrestrial methanotrophic strains, values of *K*_CH4_ are in the ppm range (Knief and Dunfield, 2005), that is, orders of magnitude higher than [CH_4_] at the surface of Mars. Because [CH_4_] << *K*_CH4_, Eq. (7) yields a (near-)linear dependence of the cell-specific CH_4_ oxidation rate, *r*, on the CH_4_ concentration. In contrast, the imposed half-saturation constant for O_2_ (1.9 ppm) is only slightly higher than the martian atmospheric O_2_ concentration and limitation by O_2_ is therefore far less severe than for CH_4_.

Based on the analysis above, we conclude that the cell-specific energy gain rates for aerobic CH_4_ oxidation in the upper martian regolith [*i.e.*, −*r* •Δ_*r*_*G*, Eq. (9)] are most likely kinetically, rather than thermodynamically, limited. Despite similar Gibbs energies of reaction, the cell-specific energy gain rates at the average mixing ratios of O_2_ and CH_4_ in Earth's atmosphere at 298.13 K and 1 atm are four orders of magnitude faster than those calculated at the default values of the mixing ratios of O_2_ and CH_4_ in the martian atmosphere at 230 K and 11 hPa. However, this conclusion depends on the extrapolation of the CH_4_ half-saturation (or affinity) constants of terrestrial methanotrophs (*K*_CH4_) to their hypothetical martian counterparts.

### 3.2. Minimum CH_4_ requirement for endogenous metabolism

**FIG. 3.**
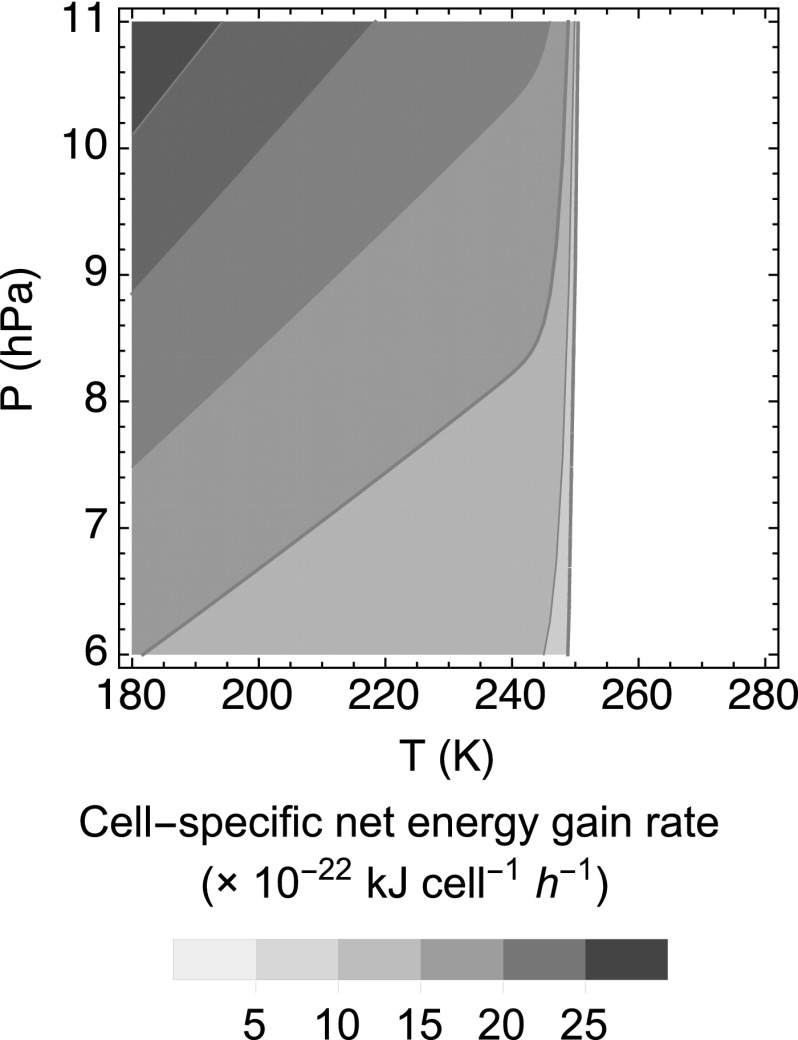
Calculated cell-specific net energy gain rate of *Methylocystis* sp. LR1 under assumed martian temperature and pressure conditions. *v_max_* and *K*_CH4_ are set to 5 × 10^−17^ mol CH_4_ cell^−1^ h^−1^ and 2.5 × 10^−6^ mol L^−1^, respectively (Knife and Dunfield, 2005); *K*_O2_ is set to 1.9 × 10^−9^ mol L^−1^ (Van Bodegom *et al.*, 2001). The cell-specific energy gain rates are calculated using Eq. (12). The imposed mixing ratios of CO_2_, O_2_, and CH_4_ are 95%, 0.145%, and 0.69 ppbv, respectively.

The cell-specific net energy gain rates for all 19 strains of terrestrial aerobic methanotrophs are estimated to be positive at <250 K (shown in Fig. 3 for *Methylocystis* sp. LR1). Although the cell-specific catabolic energy production rates of methanotrophs on Mars are predicted to be much slower than those on Earth, these rates still exceed the low metabolic energy requirement rates expected at cold temperatures. Thus, terrestrial-like aerobic methanotrophy is a potentially viable metabolism under the assumed conditions for the martian upper regolith.

**FIG. 4.**
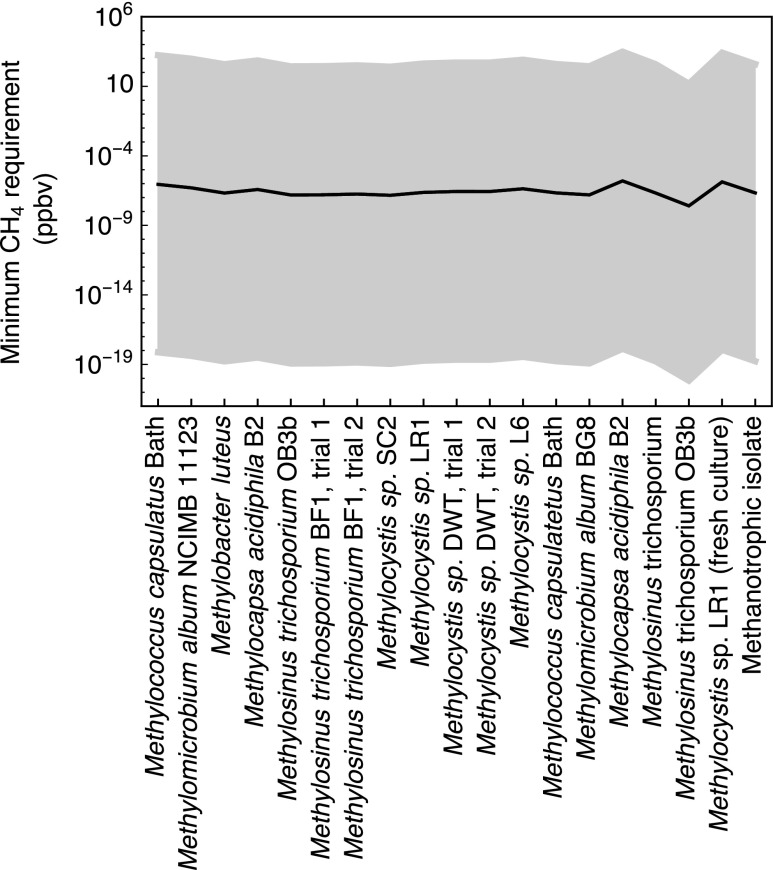
Minimum CH_4_ requirements for the 19 strains of methanotrophs at martian average temperature and pressure of 230 K and at 6 hPa; the shaded band corresponds to the values at ±30 K. The values of *v_max_* and *K*_CH4_ are extracted from the rate data for 19 strains of aerobic methanotrophs compiled by Knief and Dunfield (2005); *K*_O2_ is set to 1.9 × 10^−9^ mol L^−1^ (Van Bodegom *et al.*, 2001). The cell-specific energy gain rates are calculated with Eq. (12). The imposed mixing ratios of CO_2_, O_2_, and CH_4_ are 95%, 0.145%, and 0.69 ppbv, respectively.

The minimum CH_4_ levels needed to sustain endogenous metabolism of methanotrophs at 230 K and 6 hPa are shown in Fig. 4. At 230 K, Eq. (11) predicts a cell-specific maintenance energy requirement rate of 1.12 × 10^−27^ kJ cell^−1^ h^−1^. Together with the kinetic parameters extracted from the 19 terrestrial methanotrophic strains, the calculations then yield an average threshold CH_4_ concentration of 2.26 × 10^−6^ ppbv (maximum: 9.18 × 10^−6^ ppbv, minimum: 6.24 × 10^−8^ ppbv). According to these results, ppbv levels of CH_4_ should enable martian methanotrophs to survive. The high variability in the calculated threshold CH_4_ concentrations in Fig. 4 primarily reflects the very steep temperature dependence of the maintenance energy requirement (*m_E_*) imposed in the calculations (Fig. 1). For comparison, all other conditions unchanged, at 270 K, where *m_E_* equals 1.39 × 10^−16^ kJ cell^−1^ h^−1^, the methanotrophs would require more than 10 ppmv CH_4_ to fulfill their maintenance requirements.

### 3.3. Doubling times

**FIG. 5.**
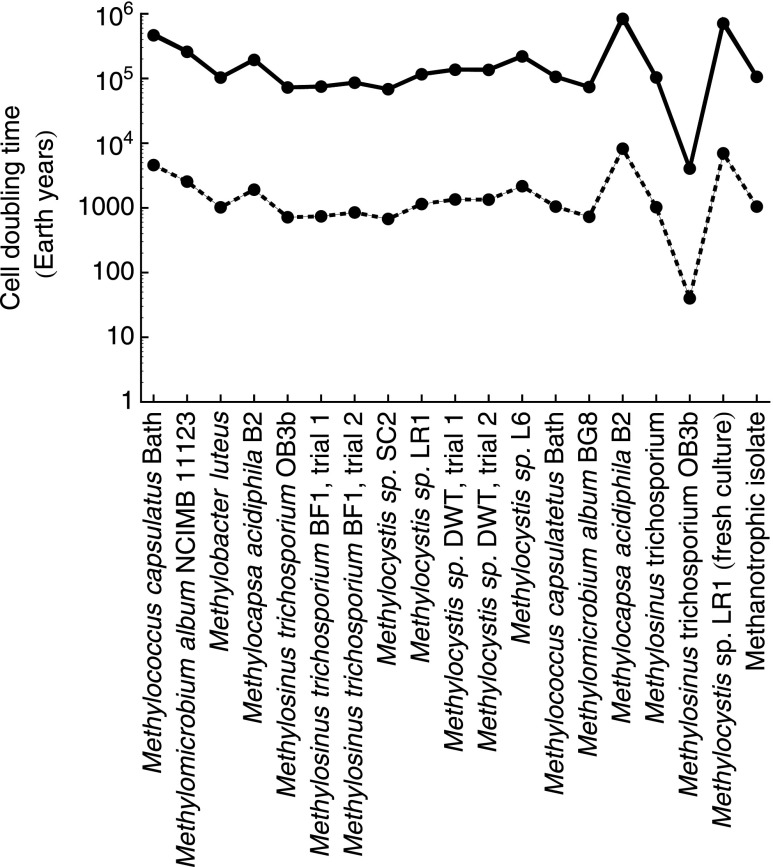
Cell doubling times for 19 strains of methanotrophs for the average of background level CH_4_ reported from Webster *et al.* (2015) (0.69 ppbv, shown by solid line), and for two orders of magnitude higher than this default value (69 ppbv, dashed line) at 230 K and 6 hPa. The values of *v_max_* and *K*_CH4_ are extracted from the rate data for 19 strains of aerobic methanotrophs compiled by Knief and Dunfield (2005); *K*_O2_ is set to 1.9 × 10^−9^ mol L^−1^ (Van Bodegom *et al.*, 2001). The cell-specific energy gain rates are calculated using Eq. (12). The imposed mixing ratios of CO_2_, O_2_, and CH_4_ are set to 95%, 0.145%, and 0.69 ppbv, respectively.

For the majority of the 19 terrestrial analog strains, Eq. (13) predicts cell doubling times in excess of 10,000 Earth years at 230 K and 0.69 ppbv CH_4_ (Fig. 5). *Methylosinus trichosporium* OB3b yields the shortest doubling time with 4831 years. The calculated doubling times for other *M. trichosporium* species are significantly longer, however, possibly reflecting the uncertainties associated with the estimation of the kinetic parameters used in Eq. (7).

The calculated cell doubling times in Fig. 5 are significantly longer than the 160 days estimated for methanotrophic bacteria retrieved from Siberian permafrost and incubated at −20°C (Rivkina *et al.*, 2000). They are also longer than the estimated generation times of subsurface cells, which are typically on the order of hundreds of years (Biddle *et al.*, 2006; Starnawski *et al.*, 2017) and occasionally up to tens of thousands of years (Jørgensen and Boetius, 2007). Similar doubling times for martian methanotrophs would require CH_4_ mixing ratios that are several orders of magnitude larger than the reported background level of 0.69 ppbv CH_4_. Even at a CH_4_ mixing ratio of 1 ppmv, which is unlikely for the martian atmosphere, the average estimated cell doubling times of the methanotrophs would still be 164 Earth years. In other words, should the methanotrophs be able to generate enough energy to survive, new biomass growth would be extremely slow. As an analogue, microorganisms retrieved from the deep subseafloor have been shown to maintain their vital functions, but only grow when newly supplied with energy sources (Morono *et al.*, 2011).

## 4. Discussion

Microbial processes account for about 69% of all CH_4_ sources on Earth (Conrad, 2009) and methanogens are among the most studied microorganisms in relation to potential life on Mars (Taubner *et al.*, 2015). The potential biogenic CH_4_ production on Mars has been estimated at 8.8 × 10^5^ molecules cm^−2^ s^−1^, based on inferred atmospheric production rates of CO and H_2_ (Krasnopolsky *et al.*, 2004). Given the reported photochemical loss rate of CH_4_ in the martian atmosphere of around 2.2 × 10^5^ molecules cm^−2^ s^−1^ (Krasnopolsky *et al.*, 2004), microbial consumption of CH_4_ could account for up to 6.6 × 10^5^ molecules cm^−2^ s^−1^ ( = 8.8 × 10^5^–2.2 × 10^5^ molecules cm^−2^ s^−1^), assuming that other CH_4_ sinks are negligible. Abiotic CH_4_ decomposition on Mars, however, could be catalyzed by meteorological-driven electric fields (Farrell *et al.*, 2006) and wind-driven erosion that produces highly reactive sites on mineral grain surfaces (Knak Jensen *et al.*, 2014).

Assuming that methanotrophs are the main CH_4_ sink, dividing the estimated 6.6 × 10^5^ molecules cm^−2^ s^−1^ consumption rate by the cell-specific rate of CH_4_ oxidation, *r* in Eq. (7), of the 19 strains, yields an average aerobic methanotrophic cell density per unit of regolith area of 1.6 × 10^6^ cells cm^−2^ (maximum: 6.5 × 10^6^ cells cm^−2^ for *Methylocapsa acidiphila* B2; minimum: 3.2 × 10^4^ cells cm^−2^ for *M. trichosporium* OB3b), at 230 K and 6 hPa, and for the default mixing ratios of O_2_ and CH_4_ in the martian atmosphere. Further assuming a carbon content for methanotrophic cells of 12 fg-C cell^−1^ (Nihous and Masutani, 2007), the average methanotrophic biomass per unit regolith surface area is then 1.9 × 10^−4^ gC m^−2^. For comparison, the average microbial biomass in the upper 0–15 cm of ocean sediment at 8 km water depth is around 0.1 gC m^−2^ (Jørgensen and Boetius, 2007). Thus, according to our results, a hypothetical methanotrophic population in martian soils would be extremely sparse.

The abundance of CH_4_ in the martian atmosphere is still under debate. Here, we use the CH_4_ mixing ratio of CH_4_ of 0.69 ppbv from the work of Webster *et al.* (2015) as the default value. The latter was recently updated to 0.41 ppbv (Webster *et al.*, 2018). For this CH_4_ level, our calculations still imply that methanotrophs should be able to support their endogenous metabolism, but that their cell doubling times would be longer (minimum: 8141 years). By contrast, other researchers have proposed CH_4_ levels of tens of ppbv (Fonti and Marzo, 2010; Geminale *et al.*, 2011), that is, significantly higher than the default value. Nonetheless, even at these higher CH_4_ mixing ratios, biomass growth of terrestrial-like methanotrophic microorganisms would remain very slow. Our calculations, however, consider a uniform CH_4_ distribution in the martian regolith. More likely, methanotrophic habitats would be concentrated near subsurface sources of CH_4_ or along preferential transport pathways, as these areas would exhibit local CH_4_ concentrations in excess of the atmospheric concentration (Webster *et al.*, 2015, 2018).

In addition to the low production and abundance of CH_4_, the metabolic activity of methanotrophs similar to that found on Earth would likely be limited by the cold surface temperatures on Mars. The lower temperature limit for terrestrial microbial metabolic activity is still an open question. Measurable metabolic activity of bacteria has been recorded for temperatures down to −20°C (Rivkina *et al.*, 2000; Junge *et al.*, 2004; Mykytczuk *et al.*, 2013), but convincing evidence for cell reproduction below −18°C is yet to be presented (Moissl-Eichinger *et al.*, 2016). Aerobic methanotrophs have been retrieved from tundra soils, polar lakes, and permafrost sediments (Trotsenko and Khmelenina, 2002, 2005). Type I methanotrophs are widely distributed in cold environments (0–10°C) (He *et al.*, 2012), and *Methylobacter*-like methanotrophs are known to be psychrophilic (Oshkin *et al.*, 2014). Viable methanotrophic bacteria from permafrost are able to oxidize CH_4_ when incubated at −5°C (Khmelenina *et al.*, 2002). Thus, although it is unclear whether permafrost bacteria are metabolically active or growing *in situ*, they retain their metabolic potential at subzero temperatures. The metabolic state of these cells therefore resembles that of microorganisms deeply buried below the seafloor, which are able to reactivate their dormant metabolic functions when re-exposed to favorable conditions (Jørgensen, 2011; Morono *et al.*, 2011).

The physiological parameters of terrestrial microorganisms that are applied under assumed martian conditions have intrinsic uncertainties associated with them. For example, the cell-specific rates of CH_4_ oxidation for the 19 strains are calculated by using just one reported value for the half-saturation constant of O_2_; *K*_O2_ = 1.9 ppm. When *K*_O2_ is three orders of magnitude higher or lower than this value, the estimated average methanotrophic microbial biomass in the martian regolith becomes 8.1 × 10^6^ or 1.6 × 10^6^ cells cm^−2^, respectively. Thus, in this particular case, the uncertainty on *K*_O2_ would actually have a relatively small influence on the predicted abundance and activity of methanotrophs.

A much higher degree of uncertainty is associated with the Arrhenius equation describing the endogenous metabolic energy requirement at temperatures below 10°C [Eq. (11)]. This equation is based on limited data that include only one aerobic bacterial species growing on methanol. The implicit assumption that microbial maintenance energy requirements are relatively insensitive to the genetic species composition is consistent with the temperature-dependent maintenance energy relationship of Tijhuis *et al.* (1993), which holds across a variety of bacterial species (Harder, 1997). Nevertheless, assessing the validity of Eq. (11) for aerobic methanotrophs surviving at extremely cold temperatures will need further data on their endogenous metabolic energy demands. In addition, the energy conversion efficiency of CH_4_ oxidation is likely temperature dependent. At extremely cold temperature, cell doubling times may become longer than the values calculated with Eq. (13), because energy dissipation as heat would be expected to increase with decreasing temperature.

The kinetic parameters for the 19 strains of terrestrial methanotrophs used to compute aerobic methane oxidation under martian conditions are based on laboratory experiments carried out under typical Earth surface temperatures and pressures. Given that for a given enzymatic reaction pathway the value of *v_max_* is expected to decrease with decreasing temperature (D'Amico *et al.*, 2006), the calculated energy gains at martian surface temperatures could be overestimated, hence compromising the proposed viability of methanotrophy in the martian regolith. For example, potential rates of CH_4_ oxidation measured in incubations with samples from Lake Qalluuraq, Alaska, show a progressive drop when decreasing the temperature from 21°C to 4°C (He *et al.*, 2012). However, because of long-term acclimation to permanently cold conditions, psychrophilic microbial populations may exhibit specific metabolic rates at low temperatures that rival those of their mesotrophic counterparts at moderate temperatures (Knoblauch and Jørgensen, 1999). To our knowledge, direct evidence of such an adaptation for psychrophilic methanotrophs is currently missing. This evidence could include measurements of relatively high methane oxidation rates at low (3–5°C) and subzero (−5°C) temperatures (Khmelenina *et al.*, 2002; Oshkin *et al.*, 2014).

Besides cold temperatures, other environmental factors, such as low pressure, high salinity, toxins, and harmful ionizing radiation, may all impede the activity of martian methanotrophs, or for that matter microbial life in general. Further studies on extremophiles would undoubtedly strengthen our ability to better account for these additional environmental stressors on the potential survival and growth of terrestrial-like microorganisms in the martian regolith.

## 5. Conclusions

Theoretical Gibbs energies of reaction, empirical maintenance energy requirements, and observed kinetic parameters for 19 strains of terrestrial methanotrophs are combined to assess thermodynamic and kinetic constraints on aerobic methanotrophy in the martian regolith. Our analysis suggests that aerobic methanotrophs similar to those found on Earth could potentially support their endogenous metabolism at the cold temperatures and ppbv CH_4_ levels encountered at the surface of Mars. Nonetheless, the calculations also imply that the low availability of CH_4_ (rather than that of O_2_) would impose a severe kinetic limitation on the cell-specific methanotrophic energy gain rates. In turn, this would result in very long cell doubling times making it unlikely that methanotrophic cells would proliferate to great numbers under current martian conditions, with the possible exception of habitats in close proximity of CH_4_ source areas or along preferential efflux pathways.

## Supplementary Material

Supplemental data

## Notes

Associate Editor: Petra Rettberg

## Abbreviation Used

ppbv: parts per billion by volume
