# Supplementary material for: Potential for Aerobic Methanotrophic Metabolism on Mars
Source: Astrobiology. 2019 Oct 3;19(10):1187–95. doi: 10.1089/ast.2018.1943 (PMC6785171; doi:10.1089/ast.2018.1943)
Supplement: Supplemental data [file Supp_Data.pdf]

Supplementary Data

Supplementary Table S1. Values for van der Waals Constants for Gases (a, b), Standard Thermodynamic Properties of Substances ($\Delta_f G^\circ$ and S°) (Lide, 2013), and Parameters Used in Calculating the Partial Molal Isobaric Heat Capacity, $C_P^{\circ *}/R = A + BT - CT^2$, at a Given Temperature (A, B, C) (Smith and Van Ness, 1987)

	a (atm L^2 mol^{-2})	b (L mol^{-1})	$\Delta_f G^\circ$ (kJ mol^{-1})	S° (J mol^{-1} K^{-1})	A (Dimensionless)	B ($\times 10^{-3}$) (K^{-1})	C ($\times 10^{-6}$) (K^{-2})
CH_4	2.271	0.0431	−50.5	186.3	1.702	9.081	−2.164
O_2	1.363	0.0319	0	205.2	3.639	0.506	0
CO_2	3.633	0.0429	−394.4	213.8	5.457	1.045	0
H_2O	—	—	−237.1	70	8.712	1.25	−0.18

Supplementary Table S2. Extracted Data from Price and Sowers (2004), Tung *et al.* (2005), and Rohde *et al.* (2008) and the Metabolic Energy Requirement Rate Shown in Fig. 1

Metabolic rate [gC $(gC)^{-1}$ h^{-1}]	*Temperature (°C)*	*Carbon source*	*Metabolic energy requirement rate* [kJ (mol C)$^{-1}$ h^{-1}]
Methanogens [points labeled B in Fig. 1 from Price and Sowers (2004)]			
5.22×10^{-5}	1	Acetate ($\gamma = 4$, $C = 2$)	2.26×10^{-2}
2.55×10^{-6}	1	Acetate ($\gamma = 4$, $C = 2$)	1.10×10^{-3}
Methanogens [solid blue diamonds in Fig. 4 from Tung *et al.* (2005)][a]			
1.52×10^{-8}	−9.4	Acetate ($\gamma = 4$, $C = 2$)	6.57×10^{-6}
4.41×10^{-9}	−11.6	Acetate ($\gamma = 4$, $C = 2$)	1.90×10^{-6}
1.39×10^{-9}	−10.0	Acetate ($\gamma = 4$, $C = 2$)	6.02×10^{-7}
Autotrophic nitrifier using CO_2 as its carbon source [points labeled K in Fig. 1 from Price and Sowers (2004)]			
1.13×10^{-12}	−40	CO_2	3.95×10^{-9}
Nitrifiers [blue triangles in Fig. 3 from Rohde *et al.* (2008)][a]			
1.82×10^{-12}	−18.3	CO_2	6.35×10^{-9}
6.72×10^{-12}	−27.5	CO_2	2.35×10^{-8}
2.57×10^{-11}	−29.3	CO_2	8.99×10^{-8}
2.87×10^{-11}	−31.4	CO_2	1.00×10^{-7}
2.17×10^{-12}	−32.2	CO_2	7.59×10^{-9}
2.35×10^{-12}	−32.2	CO_2	8.23×10^{-9}

C is the number of C-atoms of a given carbon source and γ is the degree of reduction of the C atoms in the carbon source.
[a]The units of original metabolic rates shown in these figures are gC $(gC)^{-1}$ year.

Supplementary References

Lide, D.R. (2013) *CRC Handbook of Chemistry and Physics*, 94th Edition. CRC Press, Florida, United States.

Price, P.B. and Sowers, T. (2004) Temperature dependence of metabolic rates for microbial growth, maintenance, and survival. *Proc Natl Acad Sci U S A* 101:4631–4636.

Rohde, R.A., Price, P.B., Bay, R.C., and Bramall, N.E. (2008) In situ microbial metabolism as a cause of gas anomalies in ice. *Proc Natl Acad Sci U S A* 105:8667–8672.

Smith, J.M. and Van Ness H.C. (1987) *Introduction to Chemical Engineering Thermodynamics*, 4th ed., McGraw-Hill, Singapore.

Tung, H.C., Bramall, N.E., and Price, P.B. (2005) Microbial origin of excess methane in glacial ice and implications for life on Mars. *Proc Natl Acad Sci U S A* 102:18292–18296.
